# Molecular Mechanisms of Obesity-Induced Osteoporosis and Muscle Atrophy

**DOI:** 10.3389/fphys.2016.00439

**Published:** 2016-09-29

**Authors:** Bipradas Roy, Mary E. Curtis, Letimicia S. Fears, Samuel N. Nahashon, Hugh M. Fentress

**Affiliations:** ^1^Department of Biological Sciences, Tennessee State UniversityNashville, TN, USA; ^2^Department of Agricultural and Environmental Sciences, Tennessee State UniversityNashville, TN, USA

**Keywords:** obesity, osteoporosis, muscle atrophy, IR, leptin, 5-HT, TNF-α, AGE

## Abstract

Obesity and osteoporosis are two alarming health disorders prominent among middle and old age populations, and the numbers of those affected by these two disorders are increasing. It is estimated that more than 600 million adults are obese and over 200 million people have osteoporosis worldwide. Interestingly, both of these abnormalities share some common features including a genetic predisposition, and a common origin: bone marrow mesenchymal stromal cells. Obesity is characterized by the expression of leptin, adiponectin, interleukin 6 (IL-6), interleukin 10 (IL-10), monocyte chemotactic protein-1 (MCP-1), tumor necrosis factor-alpha (TNF-α), macrophage colony stimulating factor (M-CSF), growth hormone (GH), parathyroid hormone (PTH), angiotensin II (Ang II), 5-hydroxy-tryptamine (5-HT), Advance glycation end products (AGE), and myostatin, which exert their effects by modulating the signaling pathways within bone and muscle. Chemical messengers (e.g., TNF-α, IL-6, AGE, leptins) that are upregulated or downregulated as a result of obesity have been shown to act as negative regulators of osteoblasts, osteocytes and muscles, as well as positive regulators of osteoclasts. These additive effects of obesity ultimately increase the risk for osteoporosis and muscle atrophy. The aim of this review is to identify the potential cellular mechanisms through which obesity may facilitate osteoporosis, muscle atrophy and bone fractures.

## Introduction

Obesity is a multifactorial physical abnormality that is increasing worldwide as a result of increased consumption of high-calorie diets, reduced physical activity, genetic predisposition, and excess intake of glucocorticoids (GCs) (Lee et al., 2014; Andrade et al., 2015; Karaderi et al., 2015). Recently, a number of large-scale genome-wide association studies (GWAS) have revealed the genetic architecture and biological mechanisms of obesity, and more than 250 genetic loci have been identified for monogenic, syndromic, or common forms of obesity (Karaderi et al., 2015). Despite considerable public health efforts to control obesity, the outbreak of obesity has increased over the past three decades in the United States (US). Epidemiological data suggest that nearly 36% of the US adult population is currently obese (Feresin et al., 2014). Obesity has been implicated as a potential precursor of several types of abnormalities like Type 2 diabetes mellitus (T2DM), cancer, hypertension, heart disease, osteoarthritis, cerebrovascular disease, gastrointestinal abnormalities, pulmonary abnormalities, metabolic syndrome, and dyslipidemia (Segula, 2014).

Osteoporosis is characterized by decreased bone strength and an increased risk of fractures resulting from decreased bone mass and abnormal bone quality (Jackuliak and Payer, 2014). Osteoporosis has become a significant health problem as approximately 200 million people worldwide are estimated to have osteoporosis (Roy, 2013). Decreased bone mineral density (BMD) observed in osteoporosis is associated with decaying of the microarchitecture of bone, specifically the trabecular sites including vertebrae, ribs and hips as well as the cortical sites (Roy, 2013; Feresin et al., 2014; Bala et al., 2015). Osteoporosis is initially asymptomatic at onset and is not typically diagnosed until a fracture occurs (Roy, 2013). Females are more susceptible to osteoporosis than males, although excessive use of glucocorticoids, alcoholism, cigarette smoking, low calcium intake and hypogonadism are also potential risk factors for the development of osteoporosis (Lampropoulos et al., 2012; Roy, 2013).

**Figure 1:**
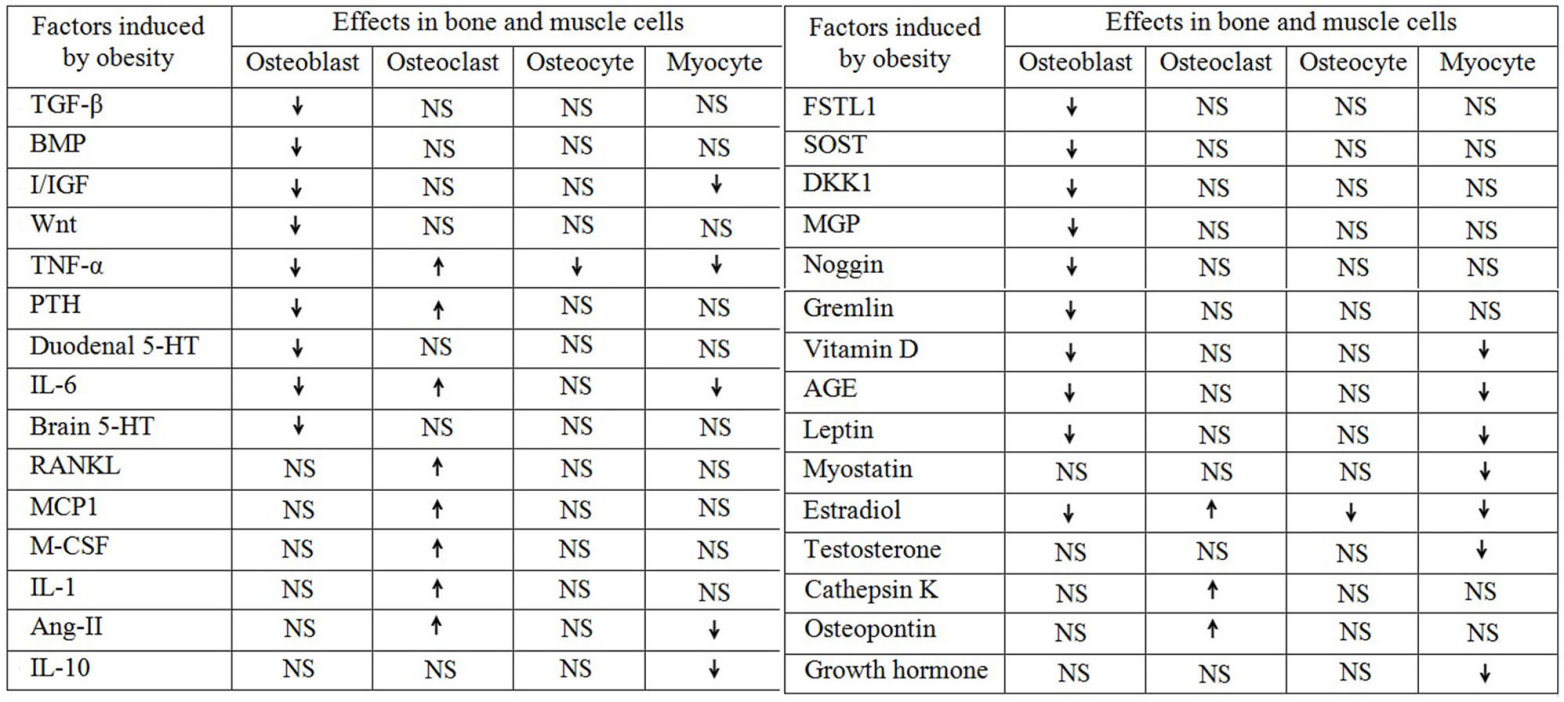
**Obesity aided regulation of different factors and their role in bone and muscle**. The up arrow “
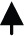
” indicates upregulation and the down arrow “
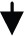
” indicates downregulation and the “NS” stands for not studied.

The incidence of osteoporosis and bone fractures has recently been shown to be more prevalent in obese individuals than individuals within a normal weight range (Halade et al., 2010; Paula and Rosen, 2010; Cao, 2011). The complete mechanism of obesity-induced osteoporosis is currently unclear, but there has been evidence that consumption of a high fat diet leading to increased total body fat mass (BFM) results in decreased BMD (Halade et al., 2010). There are several possible mechanisms through which obesity engenders the risk of osteoporosis and muscle atrophy (Figure 1). The purpose of this review is to identify cellular mechanisms through which obesity may regulate bone cells and myocytes.

## Regulators of bone formation and resorption

Osteoblasts, osteoclasts, and osteocytes are the main cells of bone (Crockett et al., 2011). Osteoblasts are non-proliferative bone-building cells that originate from osteoblast progenitor cells and aid in formation of the bone matrix by secreting osteoid, a substance responsible for bone mineralization (Dallas et al., 2013). A mature osteoblast is known as an osteocyte. Osteocytes are unable to divide and no longer secrete matrix components. Osteocytes are thought to remodel their perilacunar space, coordinate the response to mechanical loading and unloading and perform nutrient/waste exchange through blood (Lampropoulos et al., 2012; Dallas et al., 2013). Osteoclasts originate from macrophage monocyte cell lineage and participate in bone resorption, ultimately leading to decreased bone mass (Crockett et al., 2011; Lampropoulos et al., 2012).

Among the three cell types of bone, osteoblasts play the most important role in bone formation. Preosteoblasts express receptors for different types of growth factors, proinflamatory cytokines and hormones including bone morphogenetic protein (BMP), Wnt, transforming growth factor-β (TGF-β), PTH, Il-6, 5-HT, insulin/insulin like growth factor (I/IGF) and TNF (Lampropoulos et al., 2012; Roy, 2013). Binding of these ligands with their corresponding receptors induces the activation of different types of transcription factors responsible for osteoblast differentiation, maturation and survival (Lampropoulos et al., 2012). Obesity-induced adipocyte differentiation and lipid accumulation in the body has been shown to decrease the differentiation of osteoblasts since adipocytes and osteoblasts are both derived from the same mesenchymal stem cell precursors. This decrease in osteoblasts leads to decreased bone formation (Chen et al., 2010; Cao, 2011).

### Obesity aided regulation of osteoblasts

#### Obesity leads to decreased BMP signaling

**Figure 2:**
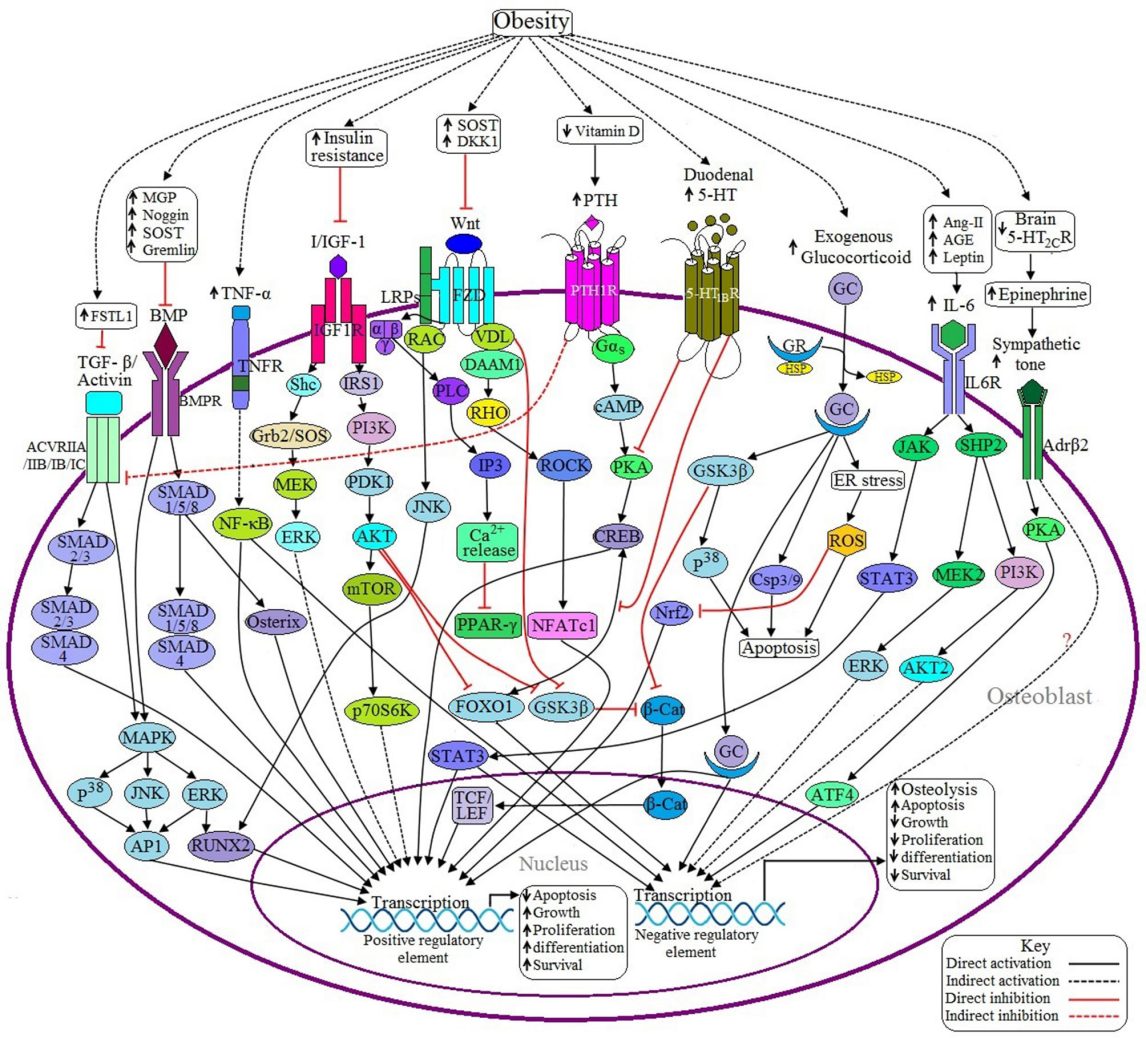
**Possible adverse effects of obesity on osteoblasts**. (a) Anabolic pathways: Binding of BMP with BMPR induces SMAD dependent and SMAD independent pathways and ultimately transcribes the genes required for osteoblast formation. In the case of SMAD dependent signaling, activation of SMAD1/5/8 recruits SMAD4 to form a SMADs complex, which in turn transcribes particular gene/genes. SMAD 1/5/8 also activates specific genes via Osterix (Osx) mediated signaling. In case of SMAD independent signaling, activated BMPR induces the transcription factor runt-related transcription factor 2 (RUNX2) and activator protein 1 (AP1) to be activated through P^38^MAPK/JNK/ERK signaling pathway. Obesity inhibits BMP signaling by upregulating the expression of some BMP inhibitors like MGP, Noggin, SOST, and Gremlin. TGF-β/Activin may activate several receptor subtypes including ACVRIIA, ACVRIIB, ACVRIB, and ACVRIC. Like BMP signaling, Activin also signals through SMAD dependent and SMAD independent pathways, but the main difference is that Activin induces SMAD2/3 and then recruits SMAD4 to form a SMADs complex. Obesity decreases Activin signaling via upregulating the expression of follistatin-like 1 (FSTL1), a potential inhibitor of Activin signaling. Binding of canonical with Frizzled/Lipoprotein receptor-related proteins (FZD/LRPs) complex activates vessel dilator (VDL), which in turns prevents β-catenin (β-cat) degradation as well as subsequent translocation of β-cat into the nucleus to activate T cell factor/lymphoid enhancer factor (TCF/LEF) by sequestering Glycogen synthase kinase 3 β (GSK3β). Binding of non-canonical Wnt with FZD triggers three different signaling pathways: (1) FZD recruits LRP and disheveled associated activator of morphogenesis 1 (DAAM1) to form a complex, which in turns activates the gene via RHO/ROCK/NFATC1 signaling pathway; (2) VDL forms a complex with Rac to activate RUNX2 via c-Jun NH_2_-terminal kinase (JNK) activation; and (3) Activated FZD induces the activation of G protein, which in turn activates Phospholipase C (PLC) to generate inositol-1,4,5-trisphosphate (IP3) to increase the cytosolic Ca^2+^ concentration and these Ca^2+^ act as negative regulators of peroxisome proliferator activated receptor γ (PPAR-γ). Obesity decreases Wnt signaling by upregulating the expression of Wnt inhibitors like SOST and Dickkopf Wnt Signaling Pathway Inhibitor 1 (DKK1). Upon activation of IGF-1 receptor (IGF-1R) by I/IGF-1 transcribes the corresponding genes through PI3K/AKT signaling and MAPK signaling pathways. Activated protein kinase B (AKT) also inhibits Forkhead box O1 (FOXO1) and GSK3β resulting in decreased expression of negative regulatory elements. Obesity-induced insulin resistance (IR) decreases I/IGF signaling. (b) Catabolic pathways: Decreased level of 5-HT from the brain suppresses bone formation by facilitating the activation of β2 adrenergic receptor (Adrβ2), which transcribes Cyclin D1 (CycD1) inhibitory factor through PKA/ATF4 signaling pathway. Adrβ2 also upregulates the expression of receptor activator of nuclear factor kappa-β ligand (RANKL) through an unknown pathway. Obesity is inversely related to brain 5-HT_2C_R expression. PTH signaling has both positive and negative impacts on osteoblast development. Activated parathyroid hormone 1 receptor (PTH1R) induces the activation of G protein, which in turn activates positive regulatory elements via cAMP/PKA/CREB signaling pathway although cAMP response element binding protein (CREB) requires the recruitment of FOXO1 to be activated. PTH1R also inhibits Activin/TGF-β signaling by inhibiting the Activin receptor (ACVR). Obesity causes the elevation of PTH via decreasing vitamin D synthesis. Duodenal 5-HT decreases PTH signaling by inhibiting protein kinase A (PKA) as well as preventing the recruitment of FOXO1 with CREB resulting in decreased osteoblastogenesis. Obesity upregulates duodenal 5-HT expression. GC binds with the glucocorticoid receptor (GR) to form the GC-GR activation complex, which induces osteoblast apoptosis through GSK3β/P^38^ signaling, generation of reactive oxygen species (ROS) by endoplasmic reticulum (ER) stress and via activation of caspase 3/9 (Csp3/9). In addition, GSK3β inhibits β-cat activity and ROS inhibits Nuclear factor-like 2 (Nrf2), a positive regulator of osteoblast. Sometimes GC activates the positive regulatory elements too. Excess GC uptake is associated with obesity. IL-6 induced JAK/STAT3 signaling has both positive and negative effects on osteoblast. In addition, IL-6 exerts its negative role via SHP2/PI3K/AKT signaling and via SHP2/MEK2/ERK signaling pathways. Obesity induces IL-6 upregulation through increased generation of Ang II, AGE and leptin. TNF-α activated TNF receptor (TNFR) activates nuclear factor-κB (NF-κB), which has both negative and positive roles in osteoblastogenesis. Obesity is responsible for increased synthesis of TNF-α.

BMPs are pleiotropic cytokines belonging to the TGF-β superfamily and are known as potent inducers of osteogenesis due to their role in inducing collagen synthesis and inhibiting collagenase-3 production (Sanchez et al., 2015). Sixteen types of BMPs have been identified and among them BMP-2, 6, and 9 may have the most significant roles in osteoblast differentiation from mesenchymal stem cells, although most BMPs are able to stimulate osteogenesis in mature osteoblasts (Cheng et al., 2003; Chen G. et al., 2012; Sanchez et al., 2015). It has also been found that BMP-2 and BMP-6 trigger osteoblast formation and chondrocyte proliferation, BMP-4 takes part in endochondral ossification, and BMP-7 induces calcium mineralization that is required for osteoblast differentiation (Chen G. et al., 2012; Zhang et al., 2013; Rasi et al., 2016). Contrarily, BMP-3 has adverse effects on osteoblastogenesis (Kokabu et al., 2012). BMP signaling has been identified as the major effector in preosteoblasts because binding of BMP with its receptor (BMPRs) activates target genes through SMAD dependent pathways or SMAD independent pathways (Chen G. et al., 2012) (Figure 2). It has recently been identified that BMP-2 exerts its effects on osteoblasts by inducing the expression of two potential osteoblastogenic factors, Phospholipase C β1 (PLCβ1) and Leucine-Rich Repeat Containing G Protein-Coupled Receptor 4 (LGR4) (Pawaputanon Na Mahasarakham et al., 2015; Ramazzotti et al., 2016). The net effect of activation of BMP signaling is to activate transcription factors necessary for the induction of osteogenesis. Matrix Gla protein (MGP), Noggin, Sclerostin (SOST), and Gremlin have been identified as several extracellular, intracellular and transcriptional BMP inhibitors that are upregulated in obesity and act as negative regulators of BMP signaling pathways, ultimately downregulating osteoblastogenesis (Lampropoulos et al., 2012; Roy, 2013). An *in vitro* study has shown that the presence of high-density lipoprotein (HDL) and low-density lipoprotein (LDL) in the body are consistent with elevated circulating MGP (Thomsen et al., 2010). Noggin is a glycosylated protein that is not only well-known for its inhibitory effects on BMP signaling pathways by sequestering the BMP ligand, but it also induces adipogenesis of mesenchymal stem cells. The association of obesity with increased Noggin levels in mesenchymal stem cells was confirmed in a preclinical, immunocompetent mouse model of spontaneous obesity and in human patients with elevated body mass index (Sawant et al., 2012). A recent study showed that pre-adipocytes are resistant to BMP4 due to increased SOST of the BMP inhibitor Gremlin 1 (Gustafson et al., 2015). Since BMP signaling is known to play an essential role in the formation of bone, BMP inhibitors will ultimately lead to decreased bone mass and increased risk of fractures.

#### Obesity decreases activin signaling via upregulation of FSTL1

Activin A is a member of the TGF-β superfamily of proteins and has been identified as a positive regulator of bone and cartilage development (Djouad et al., 2010; Lotinun et al., 2012). Knockdown of Activin A by small interfering RNA (siRNA) decreases the rate of chondrogenesis and osteogenesis from mesenchymal progenitor cells (Djouad et al., 2010). Like other TGF-β superfamily members, Activin transduces its signals through type I (ACVRIB, ACVRIC) and type II (ACVRIIA, ACVRIIB) receptor serine/threonine kinases. The binding of Activin to its receptor induces the recruitment and phosphorylation of the receptor. The activated receptor then, in turn phosphorylates SMAD2 and SMAD3 intracellular signaling proteins (Figure 2). Inhibins, FSTL1 and several other proteins have been recognized as antagonistic to Activin signaling due to their ability to interfere with Activin signaling through a variety of mechanisms (Lotinun et al., 2012). Adipose tissue of obese ob/ob mice as well as serum of overweight/obese subjects express significantly increased levels of FSTL1 (Fan et al., 2013). As FSTL1 is a known inhibitor of Activin signaling, obesity-induced increase in FSTL1 could decrease osteogenesis, leading to weakened bones and susceptibility to fractures.

#### Obesity-induced increases in DKK1 and SOST inhibit Wnt signaling

The Wnt family of highly conserved secreted glycoproteins is rich in cysteine residues and controls not only the vital developmental processes of cell fate and polarity but also some cell maintenance processes. In humans, more than 19 Wnts, 10 FZD receptors, and 2 co-receptors (LRP-5, LRP-6) have been identified so far (Tornero et al., 2015). Among several Wnt signaling pathways, the Wnt/β-cat dependent (canonical) pathway has been well-characterized (Kim J. H. et al., 2013). Binding of canonical Wnts with FZD receptors and LRP-5 or LRP-6 co-receptors promote the activation of disheveled (DVL), which in turn blocks the function of GSK3β, and prevents the degradation of β-cat as well as subsequent translocation of β-cat in the nucleus to activate the TCF/LEF family of transcription factor (Kim J. H. et al., 2013; Many and Brown, 2014) (Figure 2). Binding of non-canonical Wnts with the FZD receptor may trigger three different signaling cascades: (1) recruitment of VDL with FZD, which forms a complex with DAAM1 in order to activate the transcription factor nuclear factor of activated T-cells, cytoplasmic 1 (NFATC1) through subsequent activation of RHO and RHO associated protein kinase (ROCK); (2) VDL may form a complex with Rac to activate JNK followed by RUNX2 activation; and (3) Wnt may activate G proteins followed by the activation of phospholipase C, which catalyzes the hydrolysis of phosphatidylinositol-4,5-bisphosphate (PIP2) to IP3 and 1,2-diacylglycerol (DAG) in order to enhance the concentration of intracellular calcium ion (Ca^2+^) (Baron and Kneissel, 2013). The net effect of Wnt signaling is to inhibit the activation of negative regulatory transcription factors to allow for osteogenesis to proceed.

Obesity has been shown to increase levels of the Wnt inhibitors SOST and DKK1 (Armamento et al., 2012; Gustafson and Smith, 2012; Sheng et al., 2012). DKK1 plays a crucial role in the decrease of bone mass in obese individuals as it inhibits the Wnt signaling pathway by binding to the Kremen/LDL receptor–related protein receptors and thereby decreases the rate of osteoblast differentiation. In addition, DKK1 has been shown to increase the number of cells able to undergo adipogenesis by 3- to 4-fold, and this idea suggests that there may be a proportional relationship between obesity and DKK1 (Gustafson and Smith, 2012). Another potent inhibitor of Wnt signaling is SOST, which positively correlates with fat mass of obese individuals (Armamento et al., 2012; Sheng et al., 2012). SOST and DKK1, through their inhibition of Wnt signaling, lead to increased activation of transcription factors that act as negative regulators of osteogenesis, leading to decreased bone formation.

#### Obesity-induced insulin-resistance leads to decreased I/IGF, PI3K/AKT, and MAPK signaling

I/IGF and its downstream signaling pathways are indispensable for osteoblast development during postnatal bone growth and turnover (Pramojanee et al., 2014). The signaling pathway through which IGF exerts its effect on osteoblast is well-established. Several *in vivo* and *in vitro* studies have proposed that treatment of osteoblasts with insulin induces the activation of two signaling pathways: (1) PI3K/AKT signaling cascade via the phosphorylation of IR/IGF-1R and its downstream mediators, including insulin receptor substrate 1 (IRS-1), AKT, GSK3β, FOXO1 and p70S6K, and (2) MAPK signaling via the activation of Growth factor receptor-bound protein 2 (Grb2) (Figure 2). Activation of PI3K/AKT signaling induces osteoblast growth via upregulation of mammalian target of rapamycin C1 (mTORC1) expression, glucose uptake via upregulation of Glucose transporter type 4 (GLUT4) translocation and cell survival via downregulation of FOXO1, proapoptotic proteins like Bcl-2-associated death promoter (BAD), Bcl-2-like protein 11 (BIM) and caspases (Guntur and Rosen, 2013; Pramojanee et al., 2014). Similarly, activation of MAPK signaling induces cell proliferation via upregulation of RUNX2, Osterix and Cyc D1 and downregulation of p21cip and p27kip expression (Tahimic et al., 2013; Pramojanee et al., 2014). High fat diet induced obesity is positively correlated with IR in osteoblast and inversely related to circulating osteocalcin (OC) levels (Reinehr and Roth, 2010; Chahla et al., 2015) leading to decreased osteoblast proliferation.

#### Glucocorticoid levels seen in obesity inhibit osteoblast proliferation

Chronic or long-term exposure to GCs, as seen in obesity, inhibits osteoblast proliferation and reduces the availability of preosteoblasts by shifting the differentiation of bone marrow stromal cells (BMSCs) to favor adipocyte over osteoblast differentiation through the upregulation of adipocyte regulator CCAAT/enhancer-binding protein α (C/EBPα) (Li et al., 2013). GCs have been reported to regulate the cell cycle by decreasing the expression of Cyclin D3 (Cyc D3), the cyclic-dependent kinases (CDKs) including CDK4 and CDK6, and by increasing the transcription of CDK inhibitors such asp27 and p21 in osteosarcoma cells. GR can reduce osteoblast proliferation by suppressing CycD1 expression by binding with β-cat and by dephosphorylating extracellular signal regulated kinase (ERK) (Moutsatsou et al., 2012). GCs have also been reported to inhibit osteoblastogenesis by inhibiting RUNX2, an important osteogenic transcription factor (Rauch et al., 2010). Furthermore, GC-GR complexes also suppress the expression of OC and collagen type I through binding with negative Glucocorticoid response elements (GREs) on the OC gene promoter (Rauch et al., 2010; Li et al., 2013) and inhibit Wnt signaling by preventing translocation of β-cat into the nucleus. GC-GR complexes also induce apoptosis of osteoblasts by modulating the expression of proapoptotic and antiapoptotic genes through three different pathways: (1) GSK3β/P^38^MAPK pathway, (2) mitochondria induced Csp3/Csp9 pathway, and (3) endoplasmic reticulum (ER) stress induced ROS signaling pathways (Yun et al., 2009; Lin et al., 2014; Sato et al., 2014) (Figure 2). It is well-established that chronic use of steroid hormones, such as GCs, trigger the development of obesity accompanied by rapid bone loss (Ferris and Kahn, 2012; Lee et al., 2014).

#### Altered serotonin levels seen in obesity lead to decreased bone formation

Serotonin or 5-HT is a monoamine neurotransmitter synthesized in neurons of the brainstem and in enterochromaffin cells of the duodenum (Oury et al., 2010). Osteoblasts have been reported to express 5-HT_1A_R, 5-HT_2A_R, 5-HT_1B_R, 5-HT_2B_R, 5-HT_2C_R and 5-HT_1D_R (Ducy and Karsenty, 2010; Vernejoul et al., 2012; Dai et al., 2014) but among these subtypes, 5-HT_2A_ and 5-HT_1B_ receptors have the highest expression levels at both early and late stages of osteoblast differentiation (Dai et al., 2014). Bone mass is critically regulated by 5-HT, which exerts its functions depending on its site of synthesis (Ducy and Karsenty, 2010). Brain-derived 5-HT promotes three homeostatic functions: bone remodeling, energy expenditure and appetite, whereas duodenal-derived 5-HT inhibits osteoblast proliferation (Oury et al., 2010; Crane et al., 2015). In the brain, 5-HT binds to 5-HT_2C_R in neurons of the ventromedial hypothalamic (VMH) nuclei and inhibits the synthesis of epinephrine, thereby decreasing sympathetic tone (Oury et al., 2010) (Figure 2). This decreased sympathetic tone then, in turn, reduces the activity of β2 adrenergic receptor (β_2_AR), which positively regulates osteoblastogenesis via increased expression of CycD1 and decreased expression of RANKL through the PKA/ATF4-dependent pathway (Ducy and Karsenty, 2010). On the other hand, binding of gut-derived 5-HT to 5-HT_1B_R present on osteoblasts inhibits cAMP production as well as the phosphorylation of CREB and ultimately inhibits osteoblast proliferation (Ducy and Karsenty, 2010; Oury et al., 2010) (Figure 2). In 2012, Kode et al. proposed that the transcription factor FOXO1 is a crucial determinant of duodenal serotonin action in osteoblasts. They found that during normal levels of circulating serotonin, the proliferative activity of FOXO1 in osteoblast is controlled by a balance between its interaction with CREB and Activating transcription factor 4 (ATF4), but elevated levels of circulating serotonin prevent the interaction of FOXO1 with CREB and ultimately decrease the proliferation of osteoblasts (Kode et al., 2012). Obesity is associated with elevated peripheral serotonin and decreased levels of brain serotonin (Kim M. et al., 2013; Crane et al., 2015) and, thus, decreases bone formation by both serotonin-dependent mechanisms.

#### IL-6 is increased in obesity and antagonizes osteoblast differentiation

Overexpression of the IL-6 and IL-6 receptor (IL-6R) have been documented as antagonistic to osteoblast differentiation since IL-6 signaling strongly interferes with alkaline phosphatase (ALP) activity, which downregulates the expression of osteoblastic genes including RUNX2, Osterix, and OC, and reduces the rate of mineralization (Kaneshiro et al., 2014). Activation of IL-6R by binding of the extracellular IL-6 ligand may trigger three different signaling pathways: SHP2/MEK/ERK, SHP2/PI3K/AKT2, and JAK/STAT3 (Figure 2). The first two signaling pathways downregulate osteoblastogenesis, but the JAK/STAT3 signaling pathway acts both as a negative and positive regulator of osteoblast differentiation, leading to a net effect of decreased osteoblastogenesis. In the case of negative regulation, signal transducers and activators of transcription 3 (STAT3) inhibits MAPK signaling, while in the case of positive regulation, STAT3 acts as an osteogenic transcription factor (Kaneshiro et al., 2014; Osta et al., 2014). A recent study revealed that elevated IL-6 levels and increased expression of its soluble receptor (IL-6R) correlated positively with body mass index (BMI) and percent body fat. This finding suggests the role of obesity as a positive modulator of IL-6R and IL-6 expression in adipose tissue (Sindhu et al., 2015).

#### TNF-α is increased during obesity and inhibits osteoblast differentiation

TNF-α is another factor that has been known as an inhibitor of osteoblast differentiation and an activator of osteoclastogenesis (Osta et al., 2014). TNF-α treated pre-osteoblast (MC3T3-E1) cells show increased expression of cAMP response element-binding protein H (CREBH) by upregulating the NF-κB signaling pathway, and thereby inhibit BMP2 induced production of RUNX2, ALP, and OC (Figure 2). It has also been revealed that CREBH induces the expression of SMAD ubiquitination regulatory factor 1 (Smurf1), which in turn degrades SMAD1 via ubiquitination (Jang et al., 2015). Another study proposed that TNF-α not only induces the ubiquitination of SMADs through activating Smurf1 and Smurf2, but also upregulates DKK1 and SOST, which are potential inhibitors of BMP and Wnt signaling. Due to these effects on signaling, elevated levels of TNF-α are associated with osteoblast apoptosis (Bin et al., 2015). Decreased levels of TNF-α can favor osteogenic differentiation by upregulating the expression of BMP-2, Osx, RUNX2 and OC through a NF-κB mediated signaling pathway (Osta et al., 2014). A recent study showed that obesity mainly exerts its inhibitory effect on osteoblastogenesis by TNF-α, secreted from adipocytes (Abuna et al., 2016).

#### Obesity-induced decrease in estrogen production decreases osteoblast development

Reduced estrogen levels negatively regulate osteoblast development, but the exact mechanism is still unknown. A recent study demonstrated that estrogen signaling in osteoblasts indirectly downregulates SOST expression through interaction with BMP2-SMAD signaling pathway (Kim et al., 2015). Almeida et al. have demonstrated that estrogen receptor-α (ERα) expressed on osteoblast progenitor cells induce the expression of Osterix1 (Osx1), which potentiated Wnt/β-cat signaling, and thereby increased the proliferation and differentiation of periosteal cells (Almeida et al., 2013). It has been shown that obesity is negatively correlated with serum estradiol levels in females (Freeman et al., 2010).

#### Reduced EPCs and increased AGEs observed in obesity decrease bone strength and healing

Endothelial progenitor cells (EPCs) are bone marrow-derived stem cells, which have been recognized as angiogenic factors during bone healing (Keramaris et al., 2012; Sun et al., 2014). Obesity is associated with reduced expression of EPCs through different mechanisms and thereby decreases the rate of angiogenesis required for bone formation in the fracture sites (Chen Y. L. et al., 2012; Sun et al., 2014). Mice fed a high fat diet for 22 weeks expressed lower numbers of circulating as well as differentiated EPCs compared to mice on a high fat diet for 14 weeks (Chen Y. L. et al., 2012). Thus, not only does obesity increase the risk of fracture, but it also decreases the efficiency of fracture healing. The numbers of advanced glycation end products (AGEs) are inversely correlated with bone toughness and rigidity, due to their role in the inhibition of the synthesis of type I collagen, although the exact mechanism behind this is still unknown (Roy, 2013; Yang et al., 2014). It is now well researched that obesity is connected with increased amounts of AGE in the body (Unoki et al., 2010; Andrade et al., 2015), leading to decreased bone toughness.

### Obesity aided regulation of osteoclasts

Monocyte/macrophage cell lineage derived cells that are directly involved in osteoclastogenesis are known as osteoclasts. It is well characterized that different types of mediators such as NF-κB, RANKL, osteopontin (OPN), TNF-α, IL-6, M-CSF, and monocyte chemoattractant protein 1 (MCP1) have prominent roles to induce this process (Redlich and Smolen, 2012; Roy, 2013).

#### RANKL mediates bone resorption

**Figure 3:**
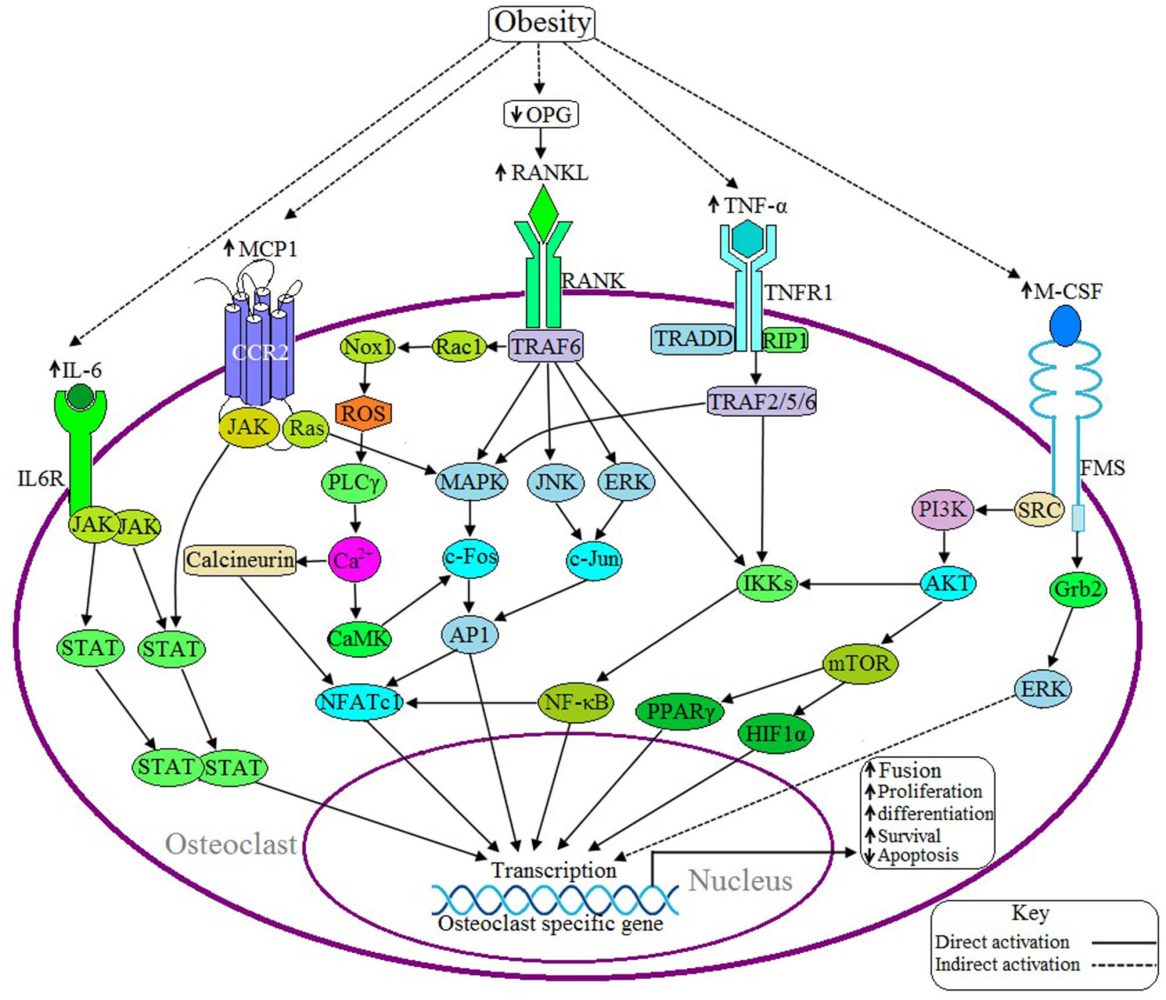
**Possible adverse effects of obesity on osteoclast**. Upon binding of RANKL with the receptor activator of nuclear factor kappa-β (RANK) activates the adaptor protein TNF receptor-associated factor 6 (TRAF6), which thereby activates the transcription factor NFATC1 via three different signaling pathways: (1) MAPK/AP1 mediated (2) IKK/NF-κB mediated, and (3) Rac/ROS/PLCγ/Ca^2+^/calcineurin mediated. Obesity induces RANKL signaling by decreasing the production of osteoprotegerin (OPG), a RANKL antagonist. IL-6 induces IL6R activation and thereby positively regulates the osteoclastic gene by the JAK/STAT signaling pathway. Binding of MCP1 with the C-C motif chemokine receptor 2 (CCR2) induces osteoclasogenesis via JAK/STAT and Ras/MAPK signaling pathways. TNF-α activates TNF receptor 1 (TNFR1) and this activation leads to recruits TNFRSF1A associated via death domain (TRADD) and Receptor-Interacting Protein 1 (RIP1) with TNFR1 and this complex thereby activates TRAF2/5/6, which in turn activates the specific gene via IKK/NF-κB and MAPK mediated signaling pathways. M-CSF activates NFATC1 through PI3K/AKT/NF-κB mediated signaling pathway or induces the activation of transcription factors peroxisome proliferator-activated receptor gamma (PPARγ) and hypoxia-inducible factor 1-alpha (HIF1α) through the PI3/AKT/mTOR signaling pathway. Obesity is associated with increased expression of IL-6, TNF-α, MCP1, and M-CSF.

RANKL is the main driver of osteoclast mediated bone resorption, and is secreted from osteoblasts and stromal cells. It functions by binding receptors expressed on the surface of cells of the monocyte/macrophage lineage and thereby induces the differentiation of pre-osteoclasts to osteoclasts via the activation of NF-κB and NFATC1 (Kim et al., 2010). RANKL inhibits osteoclast apoptosis through upregulation and expression of the antiapoptotic enzyme protein kinase B (PKB) (Figure 3) (Roy, 2013). RANKL is also implicated as a potent inducer of ROS, including free radicals, oxygen ions and peroxides, which facilitate osteoclast differentiation (Lee and Jang, 2015).

Binding of RANKL with RANK activates adaptor protein TRAF6 and subsequently activates several kinases including MAPK, JNK, and ERK. These kinases, in turn, induce the activation of NFATC1 via the activation of various hetero and homodimers of the AP1 family of proteins (Redlich and Smolen, 2012) (Figure 3). Activated TRAF6 also induces NFATC1 activation through two different signaling pathways: (1) NF-κB mediated signaling pathway and (2) Rac/ROS/PLCγ/Ca^2+^/calcineurin mediated signaling pathway (Kikuta and Ishii, 2013; Moelants et al., 2013). NFATC1 regulates the differentiation, proliferation, survival and apoptosis of osteoclasts (Kikuta and Ishii, 2013). Obesity is associated with increased production of RANKL by osteoblasts as well as elevated levels of the RANKL/OPG ratio, where OPG is an antagonist of RANKL activation (Xu et al., 2013).

In 2011, Hodge et al. found that M-CSF aids in RANKL activation because incorporation of M-CSF with RANKL accelerates the resorption processes more than with RANKL alone (Hodge et al., 2011). M-CSF has been shown to induce the activation of NFATC1 through PI3K/AKT/NF-κB mediated signaling pathway or through the activation of the transcription factors PPARγ and HIF1α through PI3K/AKT/mTOR signaling pathway (Redlich and Smolen, 2012; Kikuta and Ishii, 2013) (Figure 3). NFATC1 is an important transcription factor of osteoclastogenesis because, together, with AP1 and NF-κB, it transcribes the genes which encode the calcitonin receptor (CTR), tartrate-resistant acid phosphatase (TRAP), matrix metalloproteinase 9 (MMP9), matrix metalloproteinase 13 (MMP13), IL-6, IL-1, TNF, cathepsin K (CTSK), and carbonic anhydrase II (CA2). These proteins are required for the differentiation, proliferation, and survival, of osteoclasts as well as for acidification and degradation of bony matrix (Redlich and Smolen, 2012; Kikuta and Ishii, 2013; Moelants et al., 2013).

CTSK is a cysteine protease enzyme derived from bone resorbing macrophages and synovial fibroblasts that plays an important role in osteoclast function and in the degradation of type I and type II collagen, osteonectin and elastin which are essential bone components (Paula and Rosen, 2010). A study conducted in a mouse model found that inhibition of CTSK activity blocked the lipid accumulation in human and mouse preadipocytes. Thus, it is plausible that the serum CTSK levels are elevated in obese individuals (Yang et al., 2008), leading to increases in osteoclast proliferation.

#### Other regulators of osteoclasts

Obesity is characterized by the expression of elevated levels of proinflammatory cytokines and chemokines like TNF-α, IL-1, IL-6, and MCP-1, and these factors have adverse effects on the differentiation and activation of osteoblasts while having supportive effects on osteoclasts (Weisberg et al., 2003; Cao, 2011; Redlich and Smolen, 2012; Makki et al., 2013). In addition, obesity is associated with increased expression of leptin with decreased expression of adiponectin (Makki et al., 2013). Leptin activates β-2 adrenergic receptors on osteoblasts through the sympathetic nervous system and thereby decreases the differentiation and activation of osteoblasts while increasing the activity of osteoclasts in bone resorption. Hence, increased secretion of leptin is detrimental to bone formation while supportive for bone resorption (Ng and Duque, 2010; He et al., 2011). Adiponectin stimulates the differentiation and mineralization of the osteoblast, but directly inhibits osteoclast activity and bone resorption (Zhang et al., 2014). Ang II is another factor that positively regulates osteoclast maturation and activation. Recent studies have shown that plasma angiotensin receptor type two (AT-II) concentration increases significantly during obesity (Kotsis et al., 2010; Lampropoulos et al., 2012). OPN is an integrin binding glyco-phosphoprotein that binds with integrin receptors resulting in osteoclast activation (Lampropoulos et al., 2012). Diet induced and genetically obese mice are associated with 40- and 80-fold upregulation of OPN, respectively (Kahles et al., 2014). PTH upregulates osteoclast function, but downregulates osteoblast function and thereby facilitates bone resorption (Lampropoulos et al., 2012). A statistical analysis showed that elevated levels of PTH are more prevalent in obese people than lean people (Valina et al., 2012). ERα has been implicated as an inhibitor of osteoclast activity because Almeida et al. have shown that expression of ERα in osteoclasts prevents resorption of cancellous bone (Almeida et al., 2013).

### Obesity aided regulation of osteocytes

Osteocytes are fully mature osteoblasts that are abundantly (more than 90%) distributed throughout the mineralized bone matrix and bone surface (Bellido, 2007). Osteocytes form the lacunocanalicular network that acts as a mechanosensory system and is appropriate for mechanotransduction through which mechanical energy is transformed into biochemical signaling. Osteocyte apoptosis is very important to osteoclastogenesis because apoptotic osteocytes release immunostimulatory molecules that instruct nearby osteocytes and macrophages to produce VEGF, RANKL and other proinflamatory cytokines like TNF-α, IL-6, IL-1 (Jilka et al., 2013; Komori, 2013). Physical inactivity is a major cause of osteocyte apoptosis and it has been observed that individuals with long lasting bed rest and victims of paralysis have a greater chance of inducing osteocyte apoptosis (Bellido, 2007). Obesity is directly associated with physical immobilization (Pietilainen et al., 2008) and decreased synthesis of estrogen in the body (Freeman et al., 2010). Elevated GC levels and low-levels of estrogen are associated with higher prevalence of osteocyte apoptosis (Moutsatsou et al., 2012; Jilka et al., 2013). Reduced estrogen levels are also associated with increased production of TNF-α, that is inhibiting NO production and intracellular Ca^2+^ while strongly reducing F-actin content, resulting in decreased osteocyte stiffness followed by loss of bone mass (Osta et al., 2014). These additive effects increase osteocyte apoptosis within obese individuals.

### Obesity aided regulation of bone microcirculation

Obesity induces adipocyte differentiation from mesenchymal stem cells. The differentiated lipid cells accumulate within the bone marrow, thereby expanding the area of marrow cavity. The expanded bone marrow cavity containing bone is more susceptible to fractures than bone without an expanded cavity. The expanded bone cavity also leads to decreased bone microcirculation (Cao, 2011). Type I collagen and minerals are two important structural components of the bone tissue and the bone strength primarily depends on the quantity of collagen and BMD. An *in situ* study by Chun et al. concluded that high-fat diets acutely trigger the MMP14-dependent degradation of type I collagen fibers (Chun et al., 2010). Other studies have demonstrated a differential impact of lipid on BMD with visceral adipose tissue (VAT) having potential detrimental effects on BMD (Bredella et al., 2011). High fat diet (HFD) has a pivotal role in bone formation because it markedly reduces the rate of Ca^2+^ absorption by the intestine and thereby decreases the availability of Ca^2+^ required for osteogenesis (Xiao et al., 2010; Cao, 2011). Decreased levels of vitamin D are a hallmark of osteoporosis and bone fractures (Roy, 2013). Vitamin D deficiency in the serum prevents intestinal uptake of Ca^2+^ from the diet and thereby signals the parathyroid gland to secret increased levels of PTH. Increased secretion of PTH induces osteolysis and prevents osteogenesis by supplying adequate levels of calcium and phosphorus in the blood necessary for metabolic processes and neuromuscular function (Stojanovic et al., 2011; Binkley, 2012). Although a normal level of PTH is beneficial to bone health, elevated secretion of PTH has been identified as a negative regulator of osteoblastogenesis (Zhang et al., 2011; Lampropoulos et al., 2012). During normal levels of PTH in the blood, it binds the PTH1R and induces the expression of BMP2 via PTH1R-cAMP-PKA-CREB signaling pathway (Zhang et al., 2011). In contrast, during hyper secretion of PTH, binding of PTH with PTH1R activates PKA and ERK and then, in turn, induction of the expression of MGP on the osteoblast, which is a potent inhibitor of BMP signaling (Lampropoulos et al., 2012; Roy, 2013). PTH binding also causes the internalization of the PTH1R-TGFβR complex that sequesters TGF-β signaling in osteoblastogenesis (Chen G. et al., 2012). Vitamin D is not only playing a role in calcium and phosphorus homeostasis in the blood, but it is also required for the activation of skeletal muscle as muscle contraction is a calcium dependent process (Stojanovic et al., 2011; Binkley, 2012; Girgis et al., 2013). Obesity is a possible risk factor for decreases in vitamin D related functions because vitamin D levels are significantly lower in obese subjects compared to lean subjects and this results in an increased risk of bone fracture and osteoporosis (Segula, 2014; Pereira et al., 2015).

Beyond the effect of HFD in bone formation, HFD is also responsible for increased body weight. This excess body weight may exert its pressure on the skeletal system as well as increase the risk of falling and thereby induce the risk of fractures of the weak bones (Townsend et al., 2008; Himes and Reynolds, 2012). Several researchers have come to the conclusion that although obesity may be helpful for bone health, obesity and DM induced neuropathy may trigger osteolysis (Sinacore et al., 2008).

Systemic oxidative stress has been identified as a potential risk factor of osteoporosis and bone fractures because increased ROS due to lipid oxidation sequesters the differentiation of preosteoblasts to osteoblasts through inhibiting Wnt signaling while inducing RANKL mediated osteoclast activation (Ng and Duque, 2010; Srinivasan et al., 2010). Oxidative stress also triggers the overexpression of PPAR-γ and thereby induces the differentiation of adipocytes, rather than osteoblasts, from MSC (Ng and Duque, 2010). One study conducted on a mouse model indicated that mice deficient in superoxide dismutase (SOD) have elevated oxidative stress and decreased muscle mass and strength compared to wild-type mice (Smietana et al., 2010). Numerous studies have come to the conclusion that obese individuals contain high-levels of ROS in their adipose tissue due to over nutrition and inflammation (Krawczyk et al., 2012).

## Effect of obesity on muscle

Muscle atrophy is a physiological condition that is associated with decreased protein synthesis as well as accelerated degradation of myofibrillar and soluble proteins (Roy, 2013). Decreased protein synthesis leads to the rapid loss of skeletal muscle mass followed by weakness of muscle resulting in the increased risk of falling and ultimately bone fractures (Roy, 2013; Cohen et al., 2015). Muscle atrophy targets skeletal muscles due to denervation, fasting and various diseases like AIDS, T2DM, obesity, sepsis, renal and cardiac failure, increased GCs, trauma, and cancer. Degradation of muscle proteins takes place through two different mechanisms: ubiquitin proteosome system (UPS) and autophagy (Sala et al., 2014; Cohen et al., 2015). Myofibrillar protein components are mainly degraded by UPS, and when cellular organelles, such as mitochondria, undergo autophagy. Degradation of muscle fibers is followed by reduction of muscle strength and loss of mitochondria in myocytes due to reduced energy resulting from the deficiency of ATP synthase (Bonaldo and Sandri, 2013; Nassir and Ibdah, 2014). Muscle-specific RING-finger 1 (MuRF1) and atrogin 1 are two major ubiquitin ligases which are widely expressed in most types of atrophy (Schiaffino and Mammucari, 2011; Polge et al., 2015). MuRF1 has been identified as a potent regulator of important muscle structural proteins, including myosin heavy and light chains, troponin I, and myosin binding protein C (Bonaldo and Sandri, 2013; Schiaffino et al., 2013). Atrogin-1 induces degradation of MyoD and eukaryotic translation initiation factor 3 subunit F (eIF3f), which are important for activation of myogenic transcription factors and activation of protein synthesis, respectively (De Larichaudy et al., 2012; Lokireddy et al., 2012). Expression of both MuRF1 and atrogin-1 have been shown to be elevated in obese mice muscle compared to nonobese mice (Wang et al., 2010; Sishi et al., 2011). In addition to Murf-1 and atrogin-1, muscle atrophy is also induced by other factors including myostatin, FOXO, inducible nitric oxide synthase (iNOS), and Csp3. It has been well-established that obesity is associated with the upregulation of these factors in muscle cells as well (Perreault and Marette, 2001; Cohen et al., 2015).

Muscle growth is primarily regulated by two major signaling pathways: (1) The IGF-1–PI3K–AKT/PKB–mTOR pathway that induces protein synthesis and inhibits proteolysis and (2) The myostatin–SMAD3 pathway which inhibits protein synthesis and induces proteolysis (Egerman and Glass, 2014). Several additional pathways have also been implicated in the regulation of muscle growth. Leptin signaling has been shown to function as a positive regulator of muscle protein synthesis, whereas RAGE-MAPK, GC, Ang II, TNFR-P^38^/MAPK-NFκB, IL6-JAK-STAT signaling contribute negatively (De Larichaudy et al., 2012; Munoz-Canoves et al., 2013; Patel et al., 2014; Pellegrinelli et al., 2015). IL-10, adiponectin, and omentin have beneficial roles in muscle hypertrophy, and interferon-γ (IFN-γ) negatively regulates myogenesis, although the exact signaling pathways of these molecules in muscle cells have not been identified to date (Kim et al., 2004; Liu and Sweeney, 2014; Pelosi et al., 2014).

### Effect of I/IGF-1 on muscle

**Figure 4:**
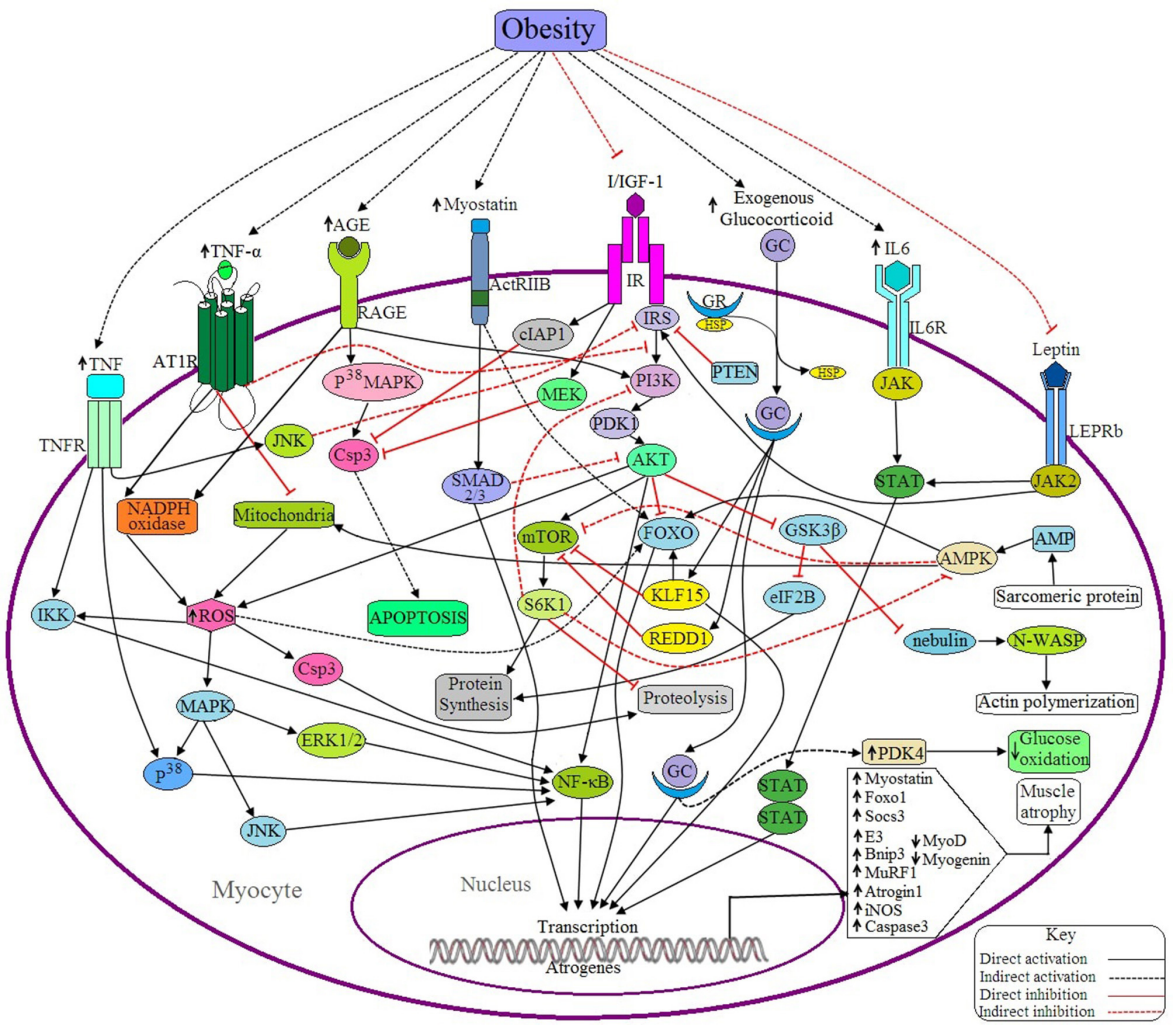
**Possible antagonistic effects of obesity on myocyte signaling to accelerate muscle atrophy**. (a) Anabolic pathways: IGF signaling is the main anabolic signaling pathway in the myocyte. Binding of I/IGF with their receptor (IR) activates IR, which in turn activates AKT through successive activations of IRS, PI3K and PDK1. Activated AKT, then in turn activates mTOR and NF-κB as well as inhibits FOXO and GSK3β. mTOR is a positive regulator of muscle because it induces the synthesis of muscle proteins and inhibits proteolysis by activating S6 kinase beta-1 (S6K1), whereas both FOXO and GSK3β are negative regulators of muscle because FOXO is a transcription factor that upregulates the transcription of E3 ubiquitin ligases and GSK3β inhibits both eIF2B and nebulin. Activated IR also inhibits Csp3 functioning through inducing the activation of cellular inhibitor of apoptosis protein 1 (cIAP1) and MAPK/ERK kinase (MEK). Leptin signaling is another anabolic signaling pathway that induces muscle protein synthesis through the JAK2/STAT signaling pathway induced by activated leptin receptor long isoform (LEPRb). Activated JAK2 may induce I/IGF signaling via the activation of IRS. (b) Catabolic pathways: Binding of GC with GR in cytosol forms the GC-GR complex and translocates into the nucleus where it induces the transcription of FOXO1, Atrogin-1, and MuRF-1 through binding with the respective genes. GC signaling also induces the upregulation of Kruppel-like factor 15 (KLF15), regulated in development and DNA damage responses -1 (REDD1) and pyruvate dehydrogenase kinase 4 (PDK4). Both KLF15 and REDD1 inhibit protein synthesis in muscle by sequestering mTOR and by inducing FOXO activation (by KLF15). PDK4 reduces glucose oxidation. IL-6 signals through the IL6R/JAK/STAT pathway and induces the overexpression of the suppressor of cytokine signaling-3 (SOCS3), a negative regulator of muscle. Myostatin induced activated ActRIIB signals through the SMAD2/3 dependent pathway and induces the expression of some proteins which downregulate the expression of MyoD and myogenin. Myostatin also induces the upregulation of atrogin1 and MuRF1 through the activation of FOXO. AGE induces the expression of Csp3 through the RAGE/P^38^MAPK signaling pathway resulting in apoptosis of myocytes. The active receptor for advanced glycation end products (RAGE) also induces the generation of ROS by activating the enzyme, NADPH oxidase. This ROS can induce the expression of Bnip3 through the activation of FOXO, and also activates NFκB via the MAPK-JNK/p^38^/ERK1/2 signaling pathway. Ang II induced activation of ATR1 triggers the generation of ROS, which in turn upregulates the expression of MuRF1 via IKK/NF-κB mediated signaling and also accelerates proteolysis via Csp3 activation. Ang II signaling downregulates IGF signaling by suppressing IRS1 and also inhibits mitochondrial biogenesis. TNF-α induces the expression of iNOS, Murf1, and Atrogin1 through NF-κB and P^38^/MAPK mediated signaling pathway. Active TNFR also inhibits IRS via JNK activation and ultimately downregulates IGF signaling. Sarcomeric protein derived AMP also activates AMPK, that in turns activates FOXO, induces mitochondrial fatty acid (FA) oxidation to generate ROS and inhibits mTOR. PTEN acts as a negative regulator of I/IGF signaling by sequestering IRS. (c) Obesity negatively regulates the anabolic pathways and positively regulates the catabolic pathways, ultimately causing muscle atrophy.

The exact role of I/IGF-1 in skeletal muscle has been extensively examined, and it was discovered that I/IGF-1 is the major protein synthesis pathway in skeletal muscle (O'Neill et al., 2015; Sharples et al., 2015) In order to determine the effect of I/IGF signaling on skeletal muscle mass, O'Neill et al. generated mice with muscle-specific knockout of IGF-1R and the insulin receptor (IR). The IGF-1R/IR knockout mice showed greater than 60% decrease in muscle mass, confirming the vital role of I/IGF signaling on skeletal muscle mass (O'Neill et al., 2015). The role of IGF-1 signaling in muscle atrophy is mainly determined by the activity of AKT, which is an intermediate signaling molecule of the IGF-1 pathway. AKT induces activation of the mammalian target of rapamycin (mTOR), which then activates S6K1, ultimately stimulating protein synthesis (Hitachi and Tsuchida, 2014) (Figure 4). AKT prevents proteolysis by repressing the FOXO family of transcription factors, and induces protein synthesis through the mTOR signaling pathway. GSK3β is a potential inhibitor of protein synthesis due to its ability to inhibit Eukaryotic translation initiation factor 2B (eIf2B) and nebulin. AKT plays a major role in muscle growth by sequestering GSK3β, thus preventing GSK3β's inhibition of protein synthesis (Schiaffino et al., 2013). It is well documented that obesity, T2DM and metabolic syndrome are associated with the progression and development of IR (Martins et al., 2012; Ruiz-Alcaraz et al., 2013). Decreased mRNA levels of IRS-1, phosphoinositide 3-kinase (PI3K), GLUT-1, GLUT-4, GSK-3 isoforms and phosphoinositide-dependent kinase-1 (PDK1) were observed in an expression study conducted on muscle samples of obese women (Colomiere et al., 2010). Similarly, reduced activation of AKT, p70S6 kinase, and mTOR were found in the skeletal muscle of obese mice and Zucker rats (Akhmedov and Berdeaux, 2013). Phosphatase and tensin homolog deleted on chromosome 10 (PTEN) is a lipid phosphatase that acts against the action of PI3K by converting phosphatidylinositol (3,4,5)-trisphosphate (PIP_3_) to PIP_2_ and preventing AKT from docking on the plasma membrane (Schiaffino et al., 2013). It has been demonstrated in mice fed a high fat diet, that PIP_3_ levels are reduced, while PTEN expression was increased (Akhmedov and Berdeaux, 2013). This observation supports the notion that obesity-induced increases in PTEN directly decrease muscle growth through the inhibition of AKT.

### Effect of myostatin on muscle

Myostatin is known as a negative regulator of myogenesis and is produced mainly by skeletal muscle, as shown by the finding that myostatin induces FOXO dependent upregulation of atrophy-related ubiquitin ligases like MuRF1 and atrogin 1 in cultured myocytes (Bonaldo and Sandri, 2013). Deactivation of the IGF-1-PI3K-AKT signaling pathway and upregulation of FOXO1 signaling is achieved by myostatin treatment (Rodriguez et al., 2014). Several studies have reported that binding of myostatin with its receptor, ActRIIB, activates SMAD 2/3 dependent signaling pathways in order to transcribe atrogenes (MacKenzie et al., 2013) (Figure 4). The proteins generated from these atrogenes inhibit the expression of MyoD and myogenin. Increased phosphorylation of SMAD2/3 proteins in obese individuals is associated with elevated levels of myostatin in skeletal muscle as well as an approximately two-fold decrease in MyoD and myogenin mRNA levels (Akhmedov and Berdeaux, 2013). Research conducted on extremely obese women determined the expression levels of myostatin within their myotubes had a 2.9-fold increase in the secretion of myostatin. From this data, it can be concluded that elevated levels of myostatin are associated with obesity (Hittel et al., 2009), and that this contributes to decreased muscle mass.

### Effect of leptin on muscle

Leptin is a 167-amino acid peptide and is the product of the ob gene that is secreted by white adipose tissue (WAT) and also by a variety of other tissues including placenta, mammary gland, ovary, skeletal muscle, stomach, pituitary gland, and lymphoid tissue (Sainz et al., 2009; Park and Ahima, 2015). Binding of leptin with its receptor (Ob-R) activates cytoplasmic tyrosine kinases of the Janus kinase family (JAKs), which then activate STAT proteins (Ceddia, 2005) (Figure 4). It has been reported that muscle mass and fiber size are significantly lower in leptin deficient ob/ob mice compared to wild type mice that were administered leptin. This data indicates that decreased leptin is associated with reduced skeletal muscle mass in leptin-deficient ob/ob mice (Sainz et al., 2009). Leptin administration in 12 week old ob/ob mice show upregulation of myocyte proliferation, elevated myogenin and myonectin transcript levels in addition to reduced mRNA expression of myostatin, dystrophin, MuRF1 and MAFbx (Rodriguez et al., 2015). Recombinant leptin bearing mice show higher expression levels of MyoD and myogenin compared to leptin receptor knockout mice. Myoblast formation rates are also higher in these mice (Arounleut et al., 2013). Both leptin and insulin resistance in myocytes are associated with the onset of obesity, type 2 diabetes, and other metabolic disorders (Yang et al., 2012). Combined, these side effects of obesity dramatically impair muscle synthesis.

### Effect of IL-6 on muscle

High doses or chronic administration of IL-6 in rats or mice cause increased degradation of proteins in skeletal muscle, although the normal levels of IL-6 have been proven hypertrophic (Munoz-Canoves et al., 2013; Pelosi et al., 2014). Insertion of the IL-6 gene into transgenic mice has been shown to elevate circulating IL-6, which is associated with severe muscle atrophy by the age of 10 weeks (Munoz-Canoves et al., 2013). Blockade of IL-6 signaling by the IL-6R antibody causes the regeneration of muscle (Carson and Baltgalvis, 2010). Indirect effects of IL-6 on IGF-1 signaling have also been reported. Increased circulating levels of IL-6 are associated with significantly reduced serum IGF-1 levels and elevated expression of SOCS3 mRNA in muscle (Munoz-Canoves et al., 2013; Pelosi et al., 2014), suggesting the role of IL-6 as a negative regulator of IGF-1 signaling. The entire mechanism by which IL-6 induces SOCS3 overexpression is still obscure, but evidence supports the hypothesis that IL-6 exerts its effect mainly through the JAK-STAT3 signaling pathway (Figure 4). In accordance with this finding, infusion of IL-6 in skeletal muscle induced STAT3 activation and overexpression of SOCS3 transcription, with a reduced number of myofibrillar proteins (Munoz-Canoves et al., 2013). Recently, the expression levels of IL-6 and its receptor, IL-6R, from lean, overweight and obese individuals were examined and it was determined that expression levels of IL-6 and IL-6R were elevated in obese individuals compared to the lean and overweight groups (Sindhu et al., 2015). A recent study showed that the overexpression of SOCS3 in skeletal muscle interferes with calcineurin signaling leading to defects in the sarcoplasmic reticulum and mitochondria. Obesity is associated with overexpression of SOCS3 in human and rodent skeletal muscle (Jorgensen et al., 2013). SOCS3 also exerts its negative effect on muscle by sequestering leptin, leading to the leptin resistance in skeletal muscle observed in obesity (Yang et al., 2012; Jorgensen et al., 2013).

### Effect of TNF-α on muscle

It has been identified from several studies that elevated levels of TNF-α are associated with increased muscle atrophy and apoptosis (Gallo et al., 2015). Obesity and T2DM are associated with a chronic inflammatory response that is characterized by increased production of TNF-α and other proinflammatory cytokines (Steinberg et al., 2006). TNF-α exerts its effects through the NF-κB and P^38^/MAPK mediated signaling pathway and stimulates the expression of iNOS, MuRF1 and MAFbx (Hall et al., 2011; Fanzani et al., 2012; Wang et al., 2014) (Figure 4). iNOS induces the production of nitric oxide (NO), which inhibits MyoD (an important myogenic transcription factor) via peroxynitrite (ONOO^−^) generation. iNOS also suppresses muscle protein synthesis through oxidative modification of JUN-D by inhibition of mTOR signaling and by increased phosphorylation of eILF2α and eukaryotic elongation factor 2 (eEF2) (Hall et al., 2011). MuRF1 and MAFbx trigger muscle protein degradation via UPS (Fanzani et al., 2012; Wang et al., 2014). TNF-α may also act as a potential inhibitor of IGF-1/AKT signaling by inhibiting the activation of IRS via JNK (Schiaffino et al., 2013). High fat diet induced obesity is associated with increased activation of JNK1 resulting in the development of obesity-induced insulin resistance (Sabio et al., 2010). Obesity is also associated with peripheral neuropathy mediated muscle atrophy or direct induction of muscle atrophy through TNF-α mediated pathways, thereby increasing muscle weakness (Sishi et al., 2011; Van et al., 2011).

### Effect of IL-10 on muscle

A knockout study in mice has shown that endogenous IL-10 attenuates IL-6 expression in skeletal muscle (Huey et al., 2008). Inhibitory effects of IL-6 on insulin signaling in skeletal muscles are reduced dramatically following treatment with IL-10 (Kim et al., 2004), showing that IL-10 acts as a negative regulator of IL-6. Reduced levels of circulating IL-10 are observed in obese muscle causing lipid-induced insulin resistance. As a result, after 3 weeks of high fat diet, mice with muscle-specific overexpression of IL-10 develop obesity, but remain insulin sensitive in skeletal muscle (Hong et al., 2009; Makki et al., 2013). The insulin sensitizing and antidiabetic effects of adiponectin are well-characterized (Liu and Sweeney, 2014). Another study implied that disruption of muscle specific AdipoR1 downregulates the adiponectin-mediated elevation of intracellular Ca^2+^ concentration, and ultimately reduced the formation of oxidative type I myofibers in skeletal muscle through the inactivation of Ca^2+^/calmodulin-dependent protein kinase (CAMK), AMP dependent protein kinase (AMPK) and Sirtuin 1 (SIRT1) (Iwabu et al., 2010). In myocytes, binding of adiponectin with its receptor (ADIPOR1/2) activates AMPK signaling and enhances fatty acid oxidation and glucose uptake in muscle (Kwon and Pessin, 2013). Obesity is characterized by the decreased generation of adiponectin in skeletal muscle and thereby, may have causal roles in mitochondrial dysfunction and insulin resistance seen in diabetic models (Iwabu et al., 2010; Makki et al., 2013). The effects of omentin on muscle cells have not been identified yet, but several studies have reported its anti-inflammatory roles in other types of cells. For example, omentin-1 triggers AKT/PKB phosphorylation and thereafter accelerates insulin induced glucose uptake in human visceral and subcutaneous adipocytes. Additionally, omentin-1 inhibits C-reactive protein (CRP) and TNF-α induced NF-κB activation in human endothelial cells (Kwon and Pessin, 2013). Lower expression levels of omentin in the serum of obese individuals have been detected and are thought to be inversely related to obesity (Duan et al., 2011; Kwon and Pessin, 2013). IFNγ/NF-κB signaling pathway has been found to induce muscle atrophy in patients affected with cancer or chronic inflammation (Di et al., 2012). Elevated expression of IFN-γ and its receptor have been observed in obese subjects with defective muscle growth (Khan et al., 2014). All of these factors contribute to decreased muscle mass.

### Effect of ectopic lipid accumulation on muscle

Obesity is characterized by ectopic lipid accumulation where elevated lipid storage takes place in subcutaneous and visceral adipose depots and non-adipose organs. There are two pools of lipids in skeletal muscles: extramyocellular lipids (EMCL) localized between muscle fibers and intramyocellular lipids (IMCL) located within muscle cells. An adipose tissue rich portion of EMCL which is closely associated with the muscle is known as intermuscular adipose tissue (IMAT). Accumulation of IMAT in obese individuals is positively correlated with insulin resistance and reduced muscle performance (Akhmedov and Berdeaux, 2013). Triacylglycerols (TAG), acyl CoAs, diacylglycerols and ceramides which are the major constituents of IMCL have been observed in higher levels in obese individuals compared to nonobese ones (Akhmedov and Berdeaux, 2013; Li et al., 2015). Lipotoxicity caused by accumulated acyl CoAs, diacylglycerols and ceramides adversely affect muscle development and regeneration (Akhmedov and Berdeaux, 2013). In obese rats and humans it was found that skeletal muscle triglycerides, diacylglycerols, and ceramides cause insulin resistance during obesity through different signaling mechanisms (Amati et al., 2011). Ceramides have also been recognized as a potential suppressor of myogenin, a transcription factor for myogenesis, as it suppresses the activity of myogenin through inhibition of phospholipase D (PLD) (Jadhav et al., 2013; Babenko and Kharchenko, 2015).

### Effect of extracellular glucose and AGE on muscle

Obesity is associated with an elevated level of extracellular glucose in the body caused by IR and this glucose is a potential precursor of AGE formation (Unoki et al., 2010; Andrade et al., 2015). Binding of AGE with its receptor, RAGE, induces the formation of ROS via the NADPH mediated signaling pathway (Daffu et al., 2013) (Figure 4). AGE is also associated with apoptosis of smooth muscle cells through expression of Csp3 via p^38^-MAPK dependent pathway (Wang et al., 2015). Overproduction of ROS causes the activation of FOXO3, a transcription factor that induces the expression of Bcl-2/adenovirus E1B 19kD-interacting protein 3 (Bnip3), which causes protein degradation, and also acts as an inhibitor of mTOR (Frost and Lang, 2011). Elevated levels of ROS may trigger muscle atrophy through three different signaling pathways: (1) the Ca^2+^ dependent calpain signaling pathway, (2) the MAPK-JNK/p^38^/ERK1/2 signaling pathway, and (3) the NF-κB signaling pathway (Fanzani et al., 2012).

### Effect of glucocorticoids on muscle

Several studies have reported the role of GCs in muscle atrophy. However, in 2013, Schakman et al. elucidated the effects of GCs on muscle atrophy by illustrating decreased fiber cross-sectional areas and reduced myofibrillar protein content. GC-induced muscle atrophy results from increased muscle proteolysis through the activation of UPS and lysosomal systems. The effect of GC on these two proteolytic systems is mediated through the upregulation of several atrogenes like FOXO, Atrogin-1, and MuRF-1 (Bonaldo and Sandri, 2013; Schakman et al., 2013; Schiaffino et al., 2013; Patel et al., 2014). GCs exert their inhibitory effects on muscle protein synthesis mainly through inhibition of the mTOR/S6 kinase 1 pathway (Schakman et al., 2013; Patel et al., 2014). REDD1 and KLF15 are two potential inhibitors of mTOR and it has been concluded that GCs induce the expression of REDD1 and KLF15 genes in skeletal muscle during atrophy (Im et al., 2007; Shimizu et al., 2011). Overexpression of REDD1 and KLF15 in skeletal muscle has been implicated during obesity (Im et al., 2007; Williamson et al., 2014). Myostatin is a negative regulator of muscle because it inhibits muscle cell proliferation and protein synthesis. It has been reported that GC treatment increases the expression of myostatin in myocytes (Patel et al., 2014). GCs further decrease glucose utilization by up regulating PDK4 expression in skeletal muscle (Figure 4), by binding with the glucocorticoid response element (GRE) since PDK4 is a suppressor of glucose oxidation (Jeong et al., 2012). Overexpression of PDK4 mRNA has been found in cultured myotubes from obese and type 2 diabetic patients (McAinch et al., 2015). The use of steroid hormone treatments in the development of different types of metabolic disorders is well-established, and the chronic use of steroid hormones, such as glucocorticoids, may trigger the development of obesity accompanied with rapid muscle atrophy (Lee et al., 2014; Patel et al., 2014).

### Effect of Angiotensin II on muscle

Ang II has been identified as antagonistic to insulin action because several lines of evidence have proven that Ang II inhibits insulin-induced tyrosine phosphorylation of IRS1, activation of AKT, and GLUT4 translocation to the plasma membrane. These actions can be reversed by pretreating myotubes with losartan (blocker of AT1 and AT2 receptor) or apocynin (a NADPH oxidase inhibitor) (Wei et al., 2006). Ang II treated C2C12 cells showed reduced expression of the genes involved in mitochondrial biogenesis and also Ang II functionality was inhibited by the blockade of the AT2 receptor (AT2R) (Mitsuishi et al., 2009). These results suggest an important role of Ang II in the inhibition of insulin signaling as well as downregulation of mitochondrial biogenesis in skeletal muscle through NADPH oxidase activation and ROS generation (Wei et al., 2006; Mitsuishi et al., 2009). Binding of Ang II with its receptor, ATR1, induces NADPH oxidase mediated generation of ROS, which in turn induces the upregulation of MuRF1 through the NF-κB signaling pathway as well as activation of Csp3, ultimately accelerating protein degradation. Ang II is also reported to induce the expression of Myostatin, GC, TNF-α and IL-6 in muscle and thereby induces muscle atrophy (Yoshida et al., 2013). In 2014, however, Oliveira et al. found that inhibition of AT1R with an AT1 receptor blocker prevented IR in rats with diet-induced obesity (Oliveira-Junior et al., 2014).

### Effect of growth hormone on muscle

Use of GH has been identified to improve physical fitness through accelerating collagen synthesis in the tendon and skeletal muscle that leads to better performance in exercise and increased muscle strength (Tavares et al., 2013). Obesity is associated with reduced levels of GH in the muscle and liver (Clasen et al., 2014). Deficiency of GH causes the reduction of muscle mass and strength and these can be reversed successfully by the supplementation of GH (Weber, 2002). GH triggers the higher expression of IGF-1 primarily of hepatic origin, although it also induces synthesis of IGF-1 in most non-hepatic tissues. A recent study showed that GH triggers STAT5b phosphorylation in muscle and fat in obese subjects along with increased expression of CISH and SOCS2, two potential cytokine inhibitors (Velloso, 2008).

### Effect of testosterone and estrogen on muscle

Testosterone and estrogen have been characterized as positive regulators of skeletal muscle mass in adult males and females respectively (Enns and Tiidus, 2010; Kovacheva et al., 2010). Testosterone exerts its effects by suppressing oxidative stress and myostatin levels, and activation of JNK and the cyclin-dependent kinase inhibitor p21 (Enns and Tiidus, 2010). During obesity, both testosterone and estrogen hormone levels decrease significantly in older males and females, respectively (Freeman et al., 2010; Wang et al., 2011). It is well-established that reduced levels of vitamin D are frequently associated with inferior physical performance and increased risk of falls due to impaired muscle activities. Depletion of vitamin D levels is a hallmark of obesity-induced muscle atrophy (Cipriani et al., 2014).

## Conclusion

**Figure 5:**
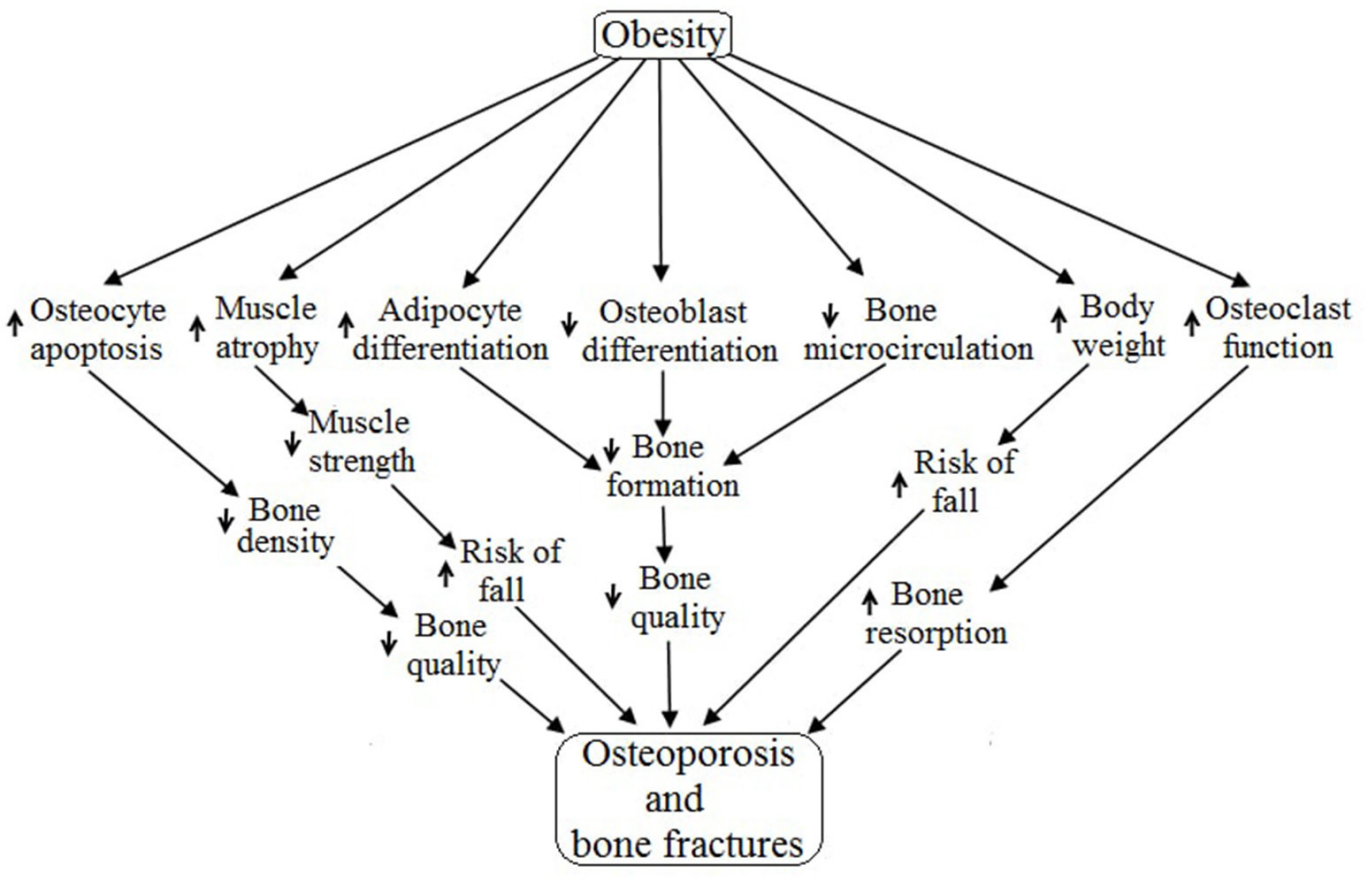
**Possible pathways of obesity-induced osteoporosis and bone fractures**.

To date, obesity has not been identified as an exact cause of osteoporosis, but it is plausible from this review to conclude that obesity may be a cause of osteoporosis and bone fractures due to its diverse range of effects on different systems of the human body. Obesity primarily regulates the skeletal system (specifically osteoblast, osteocyte, osteoclast and bone microcirculation) and the muscular system (mainly myocyte) resulting in osteoporosis as well as increased bone fractures due to decreased muscle strength (Lampropoulos et al., 2012) (Figure 5). Obesity is associated with increased expression of PTH, duodenal 5-HT, IL-6, AGEs and THF-α, as well as decreased expression of BMP, IGF, Activin/TGF-β, Wnt, brain 5-HT, estrogen and EPCs. All of these factors negatively regulate the differentiation, proliferation, survival and functioning of osteoblasts and thereby cause osteoporosis and bone fractures via reduced bone formation followed by low bone quality (Cao, 2011). Obesity is associated with exogenous GC induced apoptosis of osteocytes which result in decreased bone density followed by low bone quality, and ultimately, osteoporosis (Lee et al., 2014; Moutsatsou et al., 2012). Obesity induces the MSCs to generate more and more adipocytes rather than osteoblasts, and thereby increase bone marrow cavities followed by increases in bone fragility and decreased bone microcirculation (Cao, 2011). Obesity positively regulates osteoclasts functioning by upregulating the synthesis of RANKL, TNF-α, MCP1, IL-6, PTH and M-CSF while also downregulating ERα expression, thereby accelerating bone resorption. Obesity-induced increases in body weight escalate the chance of bone fractures by increasing the risk of fall (Valerio et al., 2012). Skeletal muscle activities are inversely related to obesity because obesity accelerates muscle atrophy and decelerates muscle strength via regulating the activities of TNF-α, Ang II, AGEs, Myostatin, exogenous GC, IL-6, I/IGF, vitamin D, Ca^2+^, estrogen, testosterone and leptin. Skeletal muscle atrophy decreases muscle strength leading to the increased risk of falls and ultimately increased chance of bone fractures.

## Author contributions

BR: Wrote down the draft and sketched the figures. MC: Reviewed and edited the manuscript. LF: Reviewed the paper. SN: Reviewed and edited the manuscript. HF: Advised, reviewed and edited the manuscript.

### Conflict of interest statement

The authors declare that the research was conducted in the absence of any commercial or financial relationships that could be construed as a potential conflict of interest.
